# An examination of the relationship among plasma brain derived neurotropic factor, peripheral vascular function, and body composition with cognition in midlife African Americans/Black individuals

**DOI:** 10.3389/fnagi.2022.980561

**Published:** 2022-08-25

**Authors:** Miranda K. Traylor, Allison J. Bauman, Napatsorn Saiyasit, Carl A. Frizell, Benjamin D. Hill, Amy R. Nelson, Joshua L. Keller

**Affiliations:** ^1^Integrative Laboratory of Exercise and Applied Physiology (iLEAP), Department of Health, Kinesiology, and Sport, College of Education and Professional Studies, University of South Alabama, Mobile, AL, United States; ^2^Department of Physiology and Cell Biology, College of Medicine, University of South Alabama, Mobile, AL, United States; ^3^Physician Assistant Sciences Program, School of Graduate Studies and Research, Meharry Medical College, Nashville, TN, United States; ^4^Department of Psychology, College of Arts and Sciences, University of South Alabama, Mobile, AL, United States

**Keywords:** health disparities, Alzheimer’s disease, exercise, brain derived neurotropic factor, cognition, body composition

## Abstract

African American/Black individuals have been excluded from several lines of prominent neuroscience research, despite exhibiting disproportionately higher risk factors associated with the onset and magnitude of neurodegeneration. Therefore, the objective of the current investigation was to examine potential relationships among brain derived neurotropic factor (BDNF), peripheral vascular function, and body composition with cognition in a sample of midlife, African American/Black individuals. Midlife adults (men: *n* = 3, 60 ± 4 years; women: *n* = 9, 58 ± 5 years) were invited to complete two baseline visits separated by 4 weeks. Peripheral vascular function was determined by venous occlusion plethysmography, a dual-energy X-ray absorptiometry was used to determine body composition, and plasma was collected to quantify BDNF levels. The CNS Vital Signs computer-based test was used to provide scores on numerous cognitive domains. The principal results included that complex attention (*r* = 0.629) and processing speed (*r* = 0.734) were significantly (*p* < 0.05) related to the plasma BDNF values. However, there was no significant (*p* > 0.05) relationship between any vascular measure and any cognitive domain or BDNF value. Secondary findings included the relationship between lean mass and peak hyperemia (*r* = 0.758) as well as total hyperemia (*r* = 0.855). The major conclusion derived from these results was that there is rationale for future clinical trials to use interventions targeting increasing BDNF to potentially improve cognition. Additionally, these results strongly suggest that clinicians aiming to improve cognitive health *via* improvements in the known risk factor of vascular function should consider interventions capable of promoting the size and function of skeletal muscle, especially in the African American/Black population.

## Introduction

African American/Black adults have historically been excluded from several lines of prominent biomedical research (Shin and Doraiswamy, 2016; Webb et al., 2022). Perhaps of greatest relevance, it was recently stated that “…most neuroscience studies recruit white participants,” (Webb et al., 2022; p. 412), which is concerning due to the known health and social disparities provoking elevated risks linked to cognitive decline (Rajan et al., 2017; Brenner and Clarke, 2018; Barnes, 2019, 2022; Wilkins et al., 2020; Saiyasit et al., 2022). Accordingly, a top public health priority includes discovering strategies to prevent, or at minimum, delay the onset of neurodegenerative conditions such as Alzheimer’s disease (AD), especially in diverse minorities (Barnes, 2019, 2022). The most common genetic risk factor for developing AD is apolipoprotein ε4 (*APOE4*) carriage (Mahley et al., 2006), which contributes to the breakdown of the human blood brain barrier (Rajan et al., 2017; Chernick et al., 2019; Montagne et al., 2020). African American/Black adults present a greater frequency of *APOE4* carriage than European Americans (Rajan et al., 2017; Alzheimer’s Association, 2022). Beyond this heightened genetic risk, it is also well documented that social, socioeconomic, and cardiovascular health status substantially contribute to the higher prevalence of conditions related to neurodegeneration in the African American/Black population (Carnethon et al., 2017; González et al., 2018; Hackman et al., 2018; Wilkins et al., 2020; Duong et al., 2021; Virani et al., 2021; Saiyasit et al., 2022). Despite advances in detecting risk factors, conditions such as vascular dysfunction and obesity still disproportionately affect this population (Carnethon et al., 2017; Brenner and Clarke, 2018; Clark et al., 2020; Virani et al., 2021). For example, Clark et al. (2020) examined midlife adults (*n* = 397; ∼17% African American) and indicated that African Americans demonstrated higher vascular risk values and lower cerebral perfusion compared to White adults. This is of considerable interest due to the necessity to maintain adequate perfusion to satisfy oxygen demands and to potentially prevent a buildup of beta-amyloid (Aβ) (Saiyasit et al., 2022). Clearly, there is an escalating need to discover meaningful outcomes, so that interventions may be designed to reverse the current public health trajectory related to neurodegeneration in the African American/Black community (Alzheimer’s Association, 2022; Saiyasit et al., 2022).

Brain derived neurotrophic factor (BDNF) is a neurotrophin that plays a significant role in regulating neuroregeneration and neuroprotection (Huuha et al., 2022). Further, it has emerged as a popular outcome variable in investigations examining the influence of exercise and/or pharmacological interventions aiming to improve brain health, likely due to its sensitivity to adjustments in modifiable risk factors and its dynamic interaction between neuro- and angiogenesis. Outside of the central nervous system (e.g., glia cells and neurons), BDNF is commonly reported to be released from skeletal and vascular smooth muscle cells as well as endothelial cells (Jodeiri Farshbaf and Alviña, 2021). In fact, a reduction in BDNF release from these cells is one of the hallmark physiological changes seen in neurodegenerative disorders, including AD (Ismail et al., 2020; Lourenco et al., 2020; Huuha et al., 2022; Oudbier et al., 2022). Increases in BDNF, however, are a hypothesized mediating pathway of cognitive enhancement following habitual and acute physical activity (Anderson-Hanley et al., 2012; Babaei et al., 2014; Fortune et al., 2019; Walsh et al., 2020; Ruiz-González et al., 2021; Umegaki et al., 2021). Thus, investigators have begun to examine the potential relationship among BDNF and cognitive function in various populations (Swardfager et al., 2011; Shimada et al., 2014; Costa et al., 2015; Fortune et al., 2019; Miranda et al., 2019; Stringham et al., 2019; Ismail et al., 2020; Lourenco et al., 2020). For example, a previous study investigated young (21.7 years), healthy males (no race/ethnicity description) and reported that there was no correlation between serum/plasma BDNF and cognitive tests (Fortune et al., 2019). In contrast, a study demonstrated that a decline in story memory and digit symbol substitution test scores was associated with BDNF levels (controlled for sex, age, education, diabetes, and smoking status) in older, community-indwelling Japanese adults (Shimada et al., 2014). Similarly, Lourenco et al. (2020) recently showed that BDNF correlated (*r*^2^ = 0.138) with mini-mental state exam scores in a sample of older Brazilian adults with and without AD. However, there remains limited available data concerning whether BDNF levels are related to cognition in African American/Black individuals, especially at midlife. It is possible that if this relationship were to be identified, it could serve as rationale for future investigations to use interventions specifically designed to increase BDNF (Walsh et al., 2020; Oudbier et al., 2022) while concurrently improving other high value correlates of BDNF such as vascular health and body composition (Gorelick et al., 2011; Gilder et al., 2014; Usui et al., 2014; González et al., 2018).

Peripheral organ health is a key factor in terms of influencing overall brain health and function (Pluvinage and Wyss-Coray, 2020; Winder et al., 2021; Huuha et al., 2022; Nelson, 2022; Oudbier et al., 2022; Saiyasit et al., 2022). In particular, numerous studies have demonstrated the importance of peripheral vascular health as it relates to the maintenance of cognitive function and prevention of neurodegeneration (Csipo et al., 2019; Albrecht et al., 2020; Winder et al., 2021; Bailey et al., 2022; Nelson, 2022; Saiyasit et al., 2022), especially given the continued increase in prevalence of modifiable risk factors like obesity that (in)directly affect vascular function (Kim and Kim, 2021; Koenen et al., 2021). Vascular dysfunction is currently the earliest detectible and fastest changing biomarker related to the onset of neurodegeneration (Iturria-Medina et al., 2016). The results of Csipo et al. (2019) have supported this by indicating that vascular-related risk factors predicted cognitive dysfunction in older adults [*n* = 29 (28, White)]. This relationship is suspected to be multifaceted, but an overall consequence of aging includes the disruption to the blood brain barrier (Chernick et al., 2019; Nation et al., 2019; Montagne et al., 2020; Pluvinage and Wyss-Coray, 2020; Nelson, 2022; Oudbier et al., 2022), which may be exacerbated by increased peripheral arterial stiffness and endothelial dysfunction (Winder et al., 2021; Bailey et al., 2022). Furthermore, a product of peripheral vascular dysfunction includes the attenuation of BDNF production considering that, as previously mentioned, BDNF is produced by the hippocampus as well as non-neuronal tissues such as vascular endothelial cells (Nakahashi et al., 2000; Lemos et al., 2016). A link between vascular-related risk factors and BDNF bioavailability has been investigated *via* the venous occlusion plethysmography (VOP) technique to reveal a relationship between BDNF and total reactive hyperemia in healthy, male Brazilian police officer recruits (Lemos et al., 2016). Mechanistically, it was proposed that various stimuli promote the release of BDNF (Fujimura et al., 2002; Lemos et al., 2016) from the endothelium, but several modifiable risk factors including obesity impair the magnitude of response resulting from shear stress. In terms of possible race/ethnic disparities, African American/Black adults exhibit worse vascular health than age-matched White adults, and perhaps as a direct consequence, the incidence of vascular related neurodegeneration is higher in African American/Black individuals compared to White adults (Gorelick et al., 2011; Carnethon et al., 2017; Clark et al., 2020; Barnes, 2022; Nelson, 2022). This disproportionate risk is likely triggered by social and biological factors that accumulate over the lifespan (Barnes, 2022; Saiyasit et al., 2022). Aligned with this notion, there is a higher reported prevalence of obesity in the African American/Black community compared to other races/ethnicities (Gorelick et al., 2011; Carnethon et al., 2017; Hales et al., 2017; Churchwell et al., 2020; Saiyasit et al., 2022), which corresponds to the relatively high reported physical *inactivity* levels (i.e., less opportunity for shear stress stimulus) in this minority group (Centers for Disease Control and Prevention, 2021). Thus, there is rationale to hypothesize that African American/Black adults may exhibit lower levels of BDNF due to greater incidences of vascular-related risk factors and obesity, which taken together, may lead to cognitive dysfunction (Saiyasit et al., 2022).

There is a persisting need to determine the best practice for improving/maintaining various tissues (adipose vs. lean tissue) across the lifespan. Several mechanisms link body composition to neurodegeneration (Gilder et al., 2014; Kaur et al., 2016; Jyväkorpi et al., 2020; Whitaker et al., 2021), and it has even been suggested that lean mass may be more related to cognitive outcomes than adipose tissue (Burns et al., 2010; Taekema et al., 2012; Spahillari et al., 2016; Mereu et al., 2018; Oudbier et al., 2022). It has been proposed that the link between lean mass and cognition includes myokine secretion (e.g., BDNF), insulin resistance, oxidative stress, and mitochondrial dysfunction (Oudbier et al., 2022). Further, recent findings indicated that Finnish adults with more favorable body composition (e.g., higher skeletal muscle mass) at midlife were associated with increased odds of reaching 90 years of age as well as higher quality of life scores at age 73 (Whitaker et al., 2021). Relatedly, lean mass is known to effect the BDNF response following exercise in young, healthy men (Gilder et al., 2014; Oudbier et al., 2022). Taken together, there is evidence to postulate that body composition is a meaningful predictor of future neurological health, and thus, encourages additional investigation, especially in diverse populations. However, it currently remains unclear, especially in the African American/Black population, whether healthcare providers should prescribe interventions focused on the reduction of fat mass or the gaining of lean mass to promote longevity *via* improvements in vascular health and body composition as it pertains to delaying neurodegeneration possibly by increasing BDNF expression (Kaur et al., 2016; Oudbier et al., 2022).

Therefore, the purpose of the current investigation was to examine potential relationships among BDNF, peripheral vascular function, and body composition with cognition in a sample of the scarcely studied midlife, African American/Black population. Based on recent studies (Mereu et al., 2018; Csipo et al., 2019; Jyväkorpi et al., 2020; Lourenco et al., 2020), it was hypothesized that cognition would be positively related to peripheral vascular function, lean body mass, and BDNF.

## Materials and methods

### Experimental design

To address our purpose, participants were invited to the Integrative Laboratory of Exercise and Applied Physiology (iLEAP) for two baseline visits separated by 4 weeks. During both visits, peripheral vascular function was assessed *via* VOP to calculate test-retest reliability. Blood pressure was also assessed with a patient monitor (Datascope Passport 2 Patient Monitor, Mindray DS USA, Inc., Mahwah, NJ, United States) at each visit. During only the first visit, body composition was quantified by a dual energy X-ray absorptiometry scan (DEXA). Additionally, plasma was collected and analyzed from this first baseline visit. In a random, counterbalanced manner, participants completed a battery of neurocognitive tests at either baseline visit one or two. Our study was not registered in a database, and all testing was conducted in a thermoneutral, ambient lit, quiet laboratory setting.

### Human participants

Twelve, midlife (50–63 years) African American/Black individuals were recruited from the local community to participate. Each individual provided signed, written informed consent prior to the initiation of any experimental procedures. Additionally, this study was performed according to the ethics standards established by the *Declaration of Helsinki* 2013 and was approved by the local Institutional Review Board for Human Subjects at the University of South Alabama (IRB#: 1760935-2) on 17 May 2021. Further, each participant provided consent for genetic testing after reviewing the Genetic Information Non-discrimination Act (GINA). No individual was diagnosed with any cognitive impairments (e.g., AD) and all participants met the inclusion criteria. Key eligibility criteria included identifying as an African American/Black man or post-menopausal woman currently aged 50–64 years old. Individuals were excluded from the study if they exhibited uncontrolled hypertension (>160/90 mmHg), neuromuscular disease, terminal illness, history of myocardial infarction, metabolic disease (e.g., diabetes), unstable cardiovascular disease, and/or musculoskeletal injury in the past 6 months. Furthermore, the purpose of this study was focused on developing rationale for future, preventative interventions to curtail neurodegeneration, thus individuals currently diagnosed or demonstrating symptoms of mild cognitive impairment or early-onset AD and related dementias were excluded.

### Venous occlusion plethysmography

The VOP procedures provided measures of peripheral vascular function. This included collecting resting forearm blood flow (FBF) by using a mercury-in-salistic strain gauge. The change in strain gauge length was recorded as voltage (EC6 Strain Gauge Plethysmograph, Hokanson Inc., WA, United States) and transmitted to a personal computer with data acquisition hardware (PowerLab 8/35SP, ADInstruments, Inc. Colorado Springs, CO, United States) and subsequently, processed as the rate (i.e., slope) of increase (LabChart Pro v8.1.16). Each strain gauge was individually chosen based upon the circumference of the widest portion (∼5 cm distal to antecubital fossa) of the right forearm of each participant. Further, while in the supine position, the right arm of the participant was abducted and supported on a portable table with an adjustable height to maintain the arm at slightly above the level of the heart. Two, independently controlled rapid inflation blood pressure cuffs were placed on the right arm: one around the uppermost portion of the arm and one around the wrist (E20 Rapid Cuff Inflator, Hokanson Inc., WA, United States). For the entirely of the VOP procedure, the wrist cuff was inflated to 200 mmHg to prevent blood from pooling in the hand vasculature. The upper arm cuff was set to automatically and rapidly inflate to a pressure of 50 mmHg and then rapidly, completely deflate. The duty cycle was set to 7 s inflated, 8 s deflated; therefore, producing one FBF value every 15 s (4 per min). To be clear, during inflation, arterial flow is minimally affected, but venous flow is occluded, causing the forearm to swell and increase the length of the strain-gauge. Values were reported as milliliters per deciliter of forearm volume (FAV) per minute (ml ⋅ dl FAV^–1^ ⋅ min^–1^). Following 3 min of resting FBF measurements (12 FBF values recorded), the upper cuff was rapidly inflated to 200 mmHg for 5 min to induce transient ischemia. Immediately following the transient ischemia, the upper arm cuff was set to cycle between inflation (4 s) and deflation (3 s) for 56 s before returning to the resting FBF procedure, 7 s:8 s. Following the completion of this reactive hyperemia phase, total hyperemia and peak FBF were calculated. Specifically, total hyperemia was simply quantified as area under the curve (i.e., summation of all FBF values greater than baseline FBF) following the 5-min of transient ischemia, whereas peak was the greatest value observed post ischemia. Additionally, resting forearm vascular conductance (FVC) was calculated *via* the following equation: [FBF ⋅ mean arterial pressure (MAP)^–1^] ⋅ 100.

### Body composition

Percent body fat, fat mass, and fat-free mass were determined from a single DEXA scan on a calibrated device (Hologic Discovery Series W, Bedford, MA, United States) based on the guidelines of the manufacturer. Height, weight, ethnicity, sex, and date of birth were entered into the DEXA software. Prior to initiating the scan, the participants removed any metal and all jewelry. Additionally, they were centered on the DEXA table in a supine position and instructed to lie still for the duration of the scan.

### Cognitive evaluation

**FIGURE 1:**
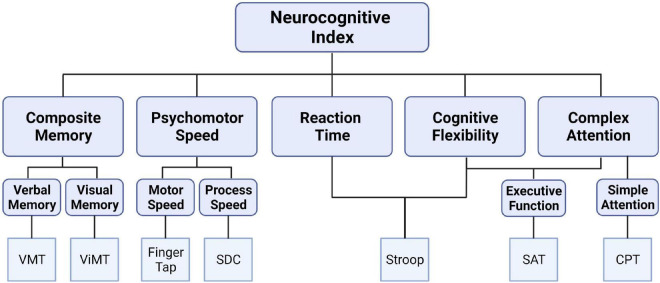
Schematic depicting the CNS Vital Signs brief-core battery testing a broad spectrum of cognitive domains from seven, discrete tests: verbal memory test (VMT), visual memory test (ViMT), finger tapping test, symbol digit coding (SDC), Stroop test, shifting attention test (SAT), and continuous performance test (CPT).

CNS Vital Signs (CNS VS; FDA Medical Device Registration Number: 3006559064) was administered on a laboratory-based computer to provide a neurocognitive assessment of a broad range of cognitive functions *via* the completion of seven, discrete tests (Figure 1). The specific tests included verbal and visual memory tests that are adaptations of the Rey Auditory Verbal Learning Test and the Rey Visual Design Learning Test, respectively. Additionally, a finger tapping test of the right and left index finders in combination with a symbol digit modalities test (variant of the Wechsler digit substitution test) was used to generate a composite score of psychomotor speed. The Stoop test was performed and consisted of three phases to quantify simple and complex reaction times. Averaging the complex reaction time scores from the Stroop test generated the domain score for “reaction time,” which is commonly referred to as “information processing speed.” A continuous performance test provided a measure of sustained attention, and the score for “complex attention” was generated by adding the number of errors committed in the continuous performance, the simple attention, and Stroop test. On average, it took the participants approximately 30 min to complete this battery of tests. The values used for analyses were the standard scores that were normalized from raw scores and present an age matched score relative to other individuals in a normative sample. Specifically, the CNS VS is standardized to have a mean score of 100 and a SD of 15 with higher scores indicating better performance.

### Venipuncture

Participants underwent venipuncture in the mornings after an overnight fast. Blood was collected into EDTA tubes (BD, Vacutainer 366643) and processed (centrifuged at ∼1,900 *g*, 22°C, 15 min). Plasma and buffy coat were aliquoted in polypropylene tubes and stored at −80°C; buffy coat was used for DNA extraction and *APOE* genotyping.

### *APOE* genotyping

DNA was extracted from buffy coat using a Research Quick-DNA Miniprep Plus Kit (Zymo Research, D4068). *APOE4* genotyping was performed using a polymerase chain reaction (PCR)-restriction fragment length polymorphism approach, as previously described (Nation et al., 2019). PCR was used to amplify a 318-base-pair fragment on a thermocycler (MiniAmp Plus Thermal Cycler, A37029, ThermoFisher) using the following primer sequences: upstream sequence (5′ ACTGACCCCGGTGGCGGAGGAGACGCGTGC) and downstream sequence (5′ TGTTCCACCAGGGGCCCCAG GCGCTCGCGG) in a 50 μl reaction. The PCR reaction mixture was incubated at 94°C for 3 min, then 40 cycles of amplification (94°C, 10 s; 65°C, 30 s; and 72°C, 30 s), then elongation at 72°C for 7 min, and then cooled to 4°C. Next restriction digests containing 10 μL of the amplicons and along with either 2.5 U *Afl*III or 1.5 U *Hae*II and incubated at 37°C overnight (15 h) in a thermocycler. *APOE* genotype was determined from the unique digestion pattern: *APOE2/2* (A: 231; H: 267); *APOE2/3* (A: 231; H: 231 and 267); *APOE2/4* (A: 231 and 295; H: 231 and 267); *APOE3/3* (A: 231; H: 231); *APOE3/4* (A: 231 and 295; H: 231); and *APOE4/4* (A: 295; H: 231); the brackets denote base pairs of amplicons following the *Afl*III (A) and *Hae*II (H) digestions.

### Plasma brain derived neurotropic factor levels

Plasma levels of BDNF were measured on the Meso Scale Discovery (MSD) platform according to the manufacturers protocol (U-PLEX Adipokine Combo 1, K15276K).

### Statistical analysis

The 2,1 model for test-retest reliability was used to evaluate intraclass correlation coefficients (ICC) and systemic error for the VOP derived outcome variables (resting FBF and FVC, peak hyperemia, and total hyperemia). These measures were then collapsed across visit, and the means were used for subsequent analyses. Pearson zero order-correlations were examined (two-tailed, *t*-test) among body composition, peripheral vascular measures (FBF, FVC, peak, and total hyperemia), BDNF, and cognitive domains. 95% confidence intervals (CI) were calculated for all significant (*p* ≤ 0.05) relationships. The statistical analyses were performed in Statistical Package for the Social Sciences (SPSS) software (version 26.0, Chicago, IL, United States).

## Results

### Subject characteristics and test-retest reliability

Three men and nine women completed this experiment. Of the 12 participants, 8 presented at least one copy of the *APOE4* allele (Table 1). The repeated measures ANOVAs used for test-retest reliability indicated moderate to strong ICCs (range = 0.43–0.95) and no significant (*p* > 0.05) systematic error. Exact *p*-values are presented in Table 2.

**TABLE 1:** Participant characteristics.

	Men (*n* = 3)	Women (*n* = 9)
Age (year)	60 ± 4	58 ± 5
Height (cm)	175.0 ± 2.5	165.3 ± 4.2
Weight (kg)	97.8 ± 14.4	91.3 ± 12.6
BMI (kg ⋅ m^–2^)	31.9 ± 3.8	33.5 ± 5.1
Lean mass (kg)	58.6 ± 1.4	43.5 ± 4.7
Body fat (%)	31.0 ± 8.2	45.9 ± 4.8
Systolic blood pressure (mmHg)	137.0 ± 4.4	139.8 ± 5.9
Diastolic blood pressure (mmHg)	86.3 ± 7.8	85.9 ± 10.4
Brain derived neurotrophic factor (pg/ml)	350.1 ± 73.9	353 ± 116.7

**TABLE 2:** Reliability (2,1 model; ICCs), systematic error (repeated measures ANOVA).

*n* = 12	Visit 1	Visit 2	ICC	*P*-Value	CV
Resting FBF	2.1 ± 0.8	2.4 ± 0.8	0.43	0.242	26.6
Resting FVC	2.0 ± 0.8	2.4 ± 0.8	0.49	0.130	26.0
Peak FBF	22.2 ± 11.6	21.3 ± 10.2	0.95	0.479	12.0
Total hyperemia	51.1 ± 26.3	48.1 ± 18.5	0.71	0.607	25.4

ANOVA, analysis of variance; CV, coefficient of variation; ICC, intraclass correlation coefficient; FBF, forearm blood flow; FVC, forearm vascular conductance.

### Brain derived neurotropic factor relationships

**FIGURE 2:**
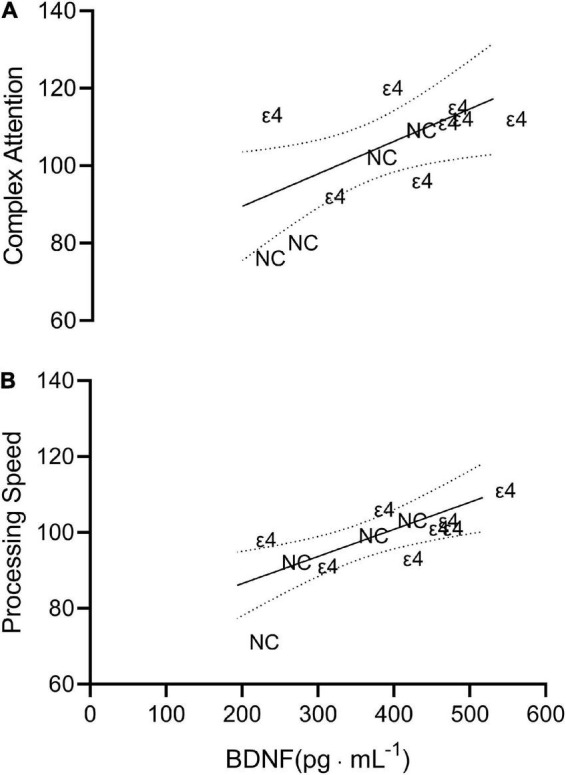
Scatterplot depicting the line of best fit plus 95% confidence interval bands for complex attention (**A**; *r* = 0.629) and processing speed (**B**; *r* = 0.734) vs. brain derived neurotropic factor (BDNF). Each individual data point is presented as either ε4 or NC indicating which data correspond to individuals with apolipoprotein ε4 carriage and non-carriers, respectively.

Complex attention (*r* = 0.629; *p* = 0.028, CI_95%_ = 0.089–0.884) and processing speed (*r* = 0.734; *p* = 0.007, CI_95%_ = 0.278–0.920) were significantly related to the plasma BDNF values as seen in Figure 2. There were no other significant (*p* > 0.05) relationships involving BDNF.

### Vascular relationships

**FIGURE 3:**
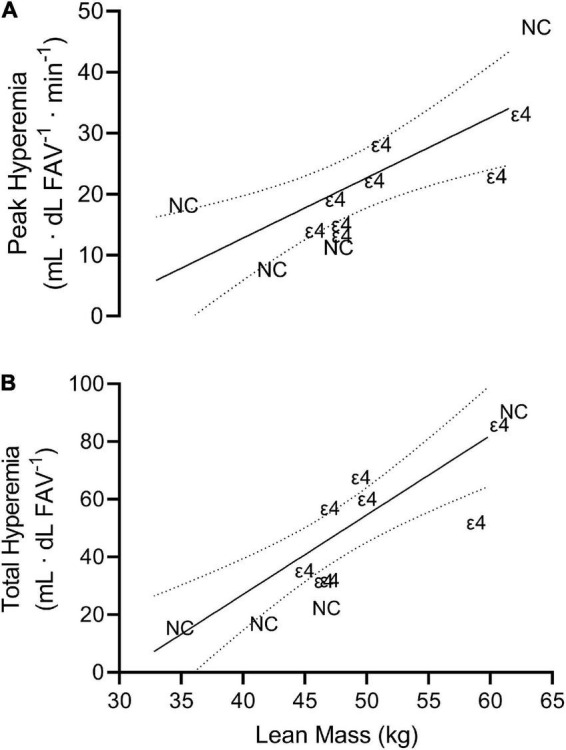
Scatterplot depicting the line of best fit plus 95% confidence interval bands for peak hyperemia (**A**; *r* = 0.758) and processing speed (**B**; *r* = 0.855) vs. lean mass. Each individual data point is presented as either ε4 or NC indicating which data correspond to individuals with apolipoprotein ε4 carriage and non-carriers, respectively.

There was no significant (*p* > 0.05) relationship between any vascular measure and any cognitive domain or BDNF value. There were, however, strong significant, positive relationships between lean mass and peak (*r* = 0.758; *p* = 0.004, CI_95%_ = 0.325–0.928) and total hyperemia (*r* = 0.855; *p* < 0.001, CI_95%_ = 0.552–0.959) FBF (Figure 3), but not resting FBF (*r* = 0.276) or FVC (*r* = 0.289). Unlike lean mass, there were no significant (*p* > 0.05) relationships among fat mass and vascular measures. Additionally, percent body fat did not significantly (*p* > 0.05) correlate with any measure of vascular function.

### Body composition relationships

Besides the above-mentioned relationships with vascular function, there were no other significant (*p* > 0.05) relationships involving lean and fat mass or percent body fat. Of possible interest, the Pearson correlation value between lean mass and composite memory was *r* = 0.500 (*p* = 0.098, CI_95%_ = −0.103 to 0.835).

### CNS Vital Signs results

The mean ± SD of each cognitive domain was dichotomized by *APOE4* status and presented in Table 3.

**TABLE 3:** Mean ± SD of each standardized cognitive domain score derived from the CNS Vital Signs evaluation dichotomized by apolipoprotein ε4 (*APOE4*) carriage status.

	*APOE4* carriage (*n* = 8)	No carriage (*n* = 4)
Neurocognition index	100.0 ± 5.7	91.8 ± 15.5
Composite memory	95.9 ± 11.4	93.3 ± 23.2
Verbal memory	95.8 ± 10.0	101.8 ± 13.8
Visual memory	97.4 ± 14.7	88.0 ± 24.9
Psychomotor speed	96.0 ± 9.0	92.0 ± 14.0
Reaction time	95.1 ± 9.7	107.8 ± 23.7
Complex attention	108.9 ± 9.7	91.8 ± 16.2
Cognitive flexibility	104.3 ± 8.5	89.5 ± 21.3
Processing speed	100.5 ± 6.5	91.3 ± 14.2
Executive function	104.0 ± 8.5	96.3 ± 13.4
Simple attention	102.0 ± 16.2	86.0 ± 26.9
Motor speed	94.5 ± 11.4	94.5 ± 9.3

## Discussion

We aimed to determine whether or not there were relationships among BDNF, peripheral vascular function, body composition, and cognition within a sample of midlife, African American/Black adults. Based on the current results, we were unable to fully support the entirety of our hypothesis. That is, our results did not indicate that body composition and vascular health were positively related to cognitive function. However, we did report that (1) two, independently calculated cognitive domains, complex attention and processing speed, were associated with plasma BDNF levels and (2) peripheral vascular function was only related to lean mass, not fat mass. Additionally, our vascular measures presented suitable reliability, supporting our below interpretations.

It has been stated that in addition to the tropic action of BDNF, it is also involved in regulating cognitive processes *via* numerous possible mechanisms including the promotion of various neurotransmitters (Miranda et al., 2019; Ismail et al., 2020; Gao et al., 2022). The current study demonstrated that the discrete complex attention (composite of the Stroop test, shifting attention test, and the continuous performance test) and processing speed (symbol digit coding test) domain scores were positively associated to plasma BDNF values in midlife African American/Black individuals. It has previously been shown that intervention-induced increases in BDNF were related to improved cognitive functioning, specifically composite memory (*r* = 0.32) and verbal memory (*r* = 0.35) in college-aged adults (Stringham et al., 2019). Like the present findings and those of Stringham et al. (2019), a positive association between BDNF and performance on neurocognitive exams assessing executive function and attention has been reported in Parkinson’s disease patients with mild cognitive impairment (Costa et al., 2015). Accordingly, these significant relationships amongst specific cognitive domains support the hypothesis that BDNF contributes to maintaining adequate frontal cortex functioning. Interestingly, age-related declines in processing speed have largely been attributed to structural changes in the prefrontal cortex. In addition to aging, other risk factors such as vascular function have been reported to be involved in the relationship between BDNF and cognition. For example, a positive association between symbol digit coding (i.e., processing speed) and BDNF was reported in Canadian patients with coronary artery disease (Swardfager et al., 2011), and notably, poorer performance on the symbol digit coding has been associated with increased risk of vascular diseases (Lai et al., 2020). Currently, however, we did not report any findings to completely support a vascular health – BDNF – cognitive function pathway in our generally, relatively healthy sample. Thus, here we can only suggest that individuals with the highest levels of resting plasma BDNF also present with the greatest functioning prefrontal cortex. It should be highlighted that the precise mediating pathway facilitating the relationship between cognitive functioning and BDNF remains to be fully elucidated. It is, however, widely agreed that BDNF is strongly associated with neuroplasticity, and thus, higher levels of this factor may enhance/maintain the function of brain regions related to attention and processing speed such as the prefrontal cortex (Anderson-Hanley et al., 2012; Miranda et al., 2019; Stringham et al., 2019; Ismail et al., 2020; Jodeiri Farshbaf and Alviña, 2021). Additionally, based on previous results (Miranda et al., 2019; Ismail et al., 2020; Lai et al., 2020), we do continue to hypothesize that, in part, BDNF elicits its proposed neurocognitive benefits in healthy individuals as well as those exhibiting signs and symptoms of neurodegeneration along the vascular neurocognitive continuum despite us not currently presenting a corresponding pathway. For instance, traditional hallmark characteristics of AD such as tau phosphorylation, Aβ accumulation, neuroinflammation, and neuronal apoptosis have previously been associated with BDNF depletion (Wang et al., 2019; Lourenco et al., 2020; Gao et al., 2022). Further, it has been suggested that increases in BDNF, perhaps *via* engagement of physical activity/exercise (Swardfager et al., 2011; Anderson-Hanley et al., 2012; Huuha et al., 2022) and subsequent reduction of vascular risk factors, promote cognitive resilience by combating these associated consequences of AD (Miranda et al., 2019; Ismail et al., 2020; Mori et al., 2021; Gao et al., 2022). In addition to AD, increased production of BDNF has been shown to benefit other neurocognitive conditions such as mild cognitive impairment (Shimada et al., 2014; Ng et al., 2021). Specifically, Shimada et al. (2014) demonstrated that within a large, Japanese older adult sample low levels of serum BDNF were a significant risk factor for the development of mild cognitive impairment. Despite these previous reports and the current results (especially considering the current sample size), there remains a critical need to extend previous findings to the African American/Black community (Shimada et al., 2014; Lourenco et al., 2020; Ng et al., 2021; Gao et al., 2022; Huuha et al., 2022). The current results, however, were encouraging and do support that developing interventions promoting BDNF *may* offer therapeutic benefits to individuals currently experiencing or at risk of neurodegenerative-related symptoms. Therefore, at minimum, our findings that BDNF levels in midlife African American/Black individuals were positively associated to cognitive abilities linked to the frontal cortex can be used to inform the direction of future, clinical trials, especially those that aim to study this population.

To our surprise, neither fat mass nor lean mass was related to any marker of cognitive function. Our original hypothesis was partially based on the notion that AD patients typically present with lower lean mass and higher body fat percentages compared to healthy controls, as well as that cognitive decline likely precedes muscle loss (Taekema et al., 2012; Mereu et al., 2018). Additionally, data from the British Regional Heart Study has suggested that individuals in the uppermost quintile of total fat mass were most likely to present with severe cognitive impairment even after adjusting for risk factors such as alcohol consumption, smoking, physical activity, history of CVD, and inflammatory markers (Papachristou et al., 2015). Mechanistically, it has been proposed that excessive adiposity is linked to poor cognitive function *via* insulin resistance (e.g., impaired glucose transport across the blood brain barrier) (Luchsinger and Gustafson, 2009). Thus, a possible explanation as to why the current study did not observe a body composition – cognitive relationship is likely due to our exclusion criteria pertaining to diabetes. That is, since the current participants did not report any form of diabetes, it is possible that they have not experienced the neurocognitive declines associated with insulin resistance (Luchsinger and Gustafson, 2009; Papachristou et al., 2015). Furthermore, Kaur et al. (2016) also examined midlife adults (*n* = 3, African Americans) to propose that the BDNF pathway is likely a contributing mechanism by which excessive adiposity impairs cognitive function, and that this too is influenced by insulin resistance. Collectively, it is our interpretation that we did not observe any relationship between body composition values and cognitive scores or BDNF due to not studying individuals presenting with insulin resistance.

The current results indicated that there were strong, positive relationships between lean mass and peripheral vascular function (i.e., peak and total flow following transient ischemia), yet fat mass was not found to correlate with any vascular measure. Of note, we quantified vascular function by inducing transient ischemia and measuring the related endothelial-dependent vasodilation known to be triggered in large part by the release of nitric oxide (NO). It has been well-documented that a benefit of habitual physical activity is the shear stress induced by repeated bouts of increased muscle blood flow, provoking an upregulation of NO. This upregulation may even reduce the magnitude of NO degradation by free radicals, decrease free radical production, and increase antioxidants, all of which contribute to greater vasodilation (Green et al., 2008). Taken together, here it is assumed that even though the current participants were screened using the same in/exclusion criteria, it is likely that there was heterogeneity in their physical activity history such that the individuals with lifestyles that include more physical activity likely presented with greater amounts of lean mass, and consequently, the greatest peripheral vascular function. Although, it is interesting that we did not find a corresponding relationship between fat mass and vascular function (e.g., more fat and worse vascular health), especially since previous reports have presented a link between obesity and vascular health (Pierce et al., 2009). However, there have been conflicting reports that this link is not directly mediated by excess fat mass, but rather coexisting conditions, such as obstructive sleep apnea, that reduce NO availability and increases oxidative stress (Jelic et al., 2010). In agreement, greater lean mass, not less fat mass, is inversely associated with all-cause and cardiovascular-specific mortality (Spahillari et al., 2016). Therefore, based on our current findings as well as previous reports (Jelic et al., 2010; Spahillari et al., 2016), we postulate that losing skeletal muscle mass may be more disadvantageous to healthy aging compared to the accumulation of fat mass, and thus, future clinical trials should *primarily* focus on the promotion of lean mass (e.g., resistance training), not the reduction of fat mass.

The current experiment is not without limitations and readers should consider the following aspects when evaluating our above interpretations. Firstly, our sample size was not large enough to determine *APOE4*- or sex-specific relationships. Additionally, based on the assumed heterogeneity resulting from the size of the sample, it is possible that some cognitive relationships may have been undetected. Furthermore, we aimed to describe the association between plasma BDNF and cognitive performance, not a causal relationship. Thus, causal statements should not be formed based on the presented data. We did not assess individual polymorphism characteristics of the BDNF gene (e.g., Val66Met), which may have offered additional insights and interpretations. Additionally, we did not use the HOMA-IR tool, which, in theory, would have strengthened some of the above conclusions. For example, had this assessment tool been utilized, descriptive information related to insulin resistance could have been provided on a participant-by-participant basis. A notable strength of the study, however, was the inclusion of test-retest reliability statistics related to the vascular function values. Accordingly, as seen in Table 2, we presented suitable ICC (0.43–0.95) while also not reporting any significant mean differences in baseline values (repeated measures ANOVA; *p* > 0.05).

## Conclusion

In summary, here for the first time we presented data to demonstrate that plasma BDNF levels are related to cognitive function in midlife, African Americans/Black individuals. Additionally, the current results indicated that peripheral vascular function was only related to lean mass, not fat mass. As previously mentioned, there is a clear lack of research focusing on racial and ethnic disparities in biomedical sciences, especially in areas including, but not limited to, the underlying pathophysiology of neurodegenerative diseases like AD (e.g., BDNF release). Thus, the current findings may be used to partially bridge the gap in available data within this population as well as be used to inform the direction of future clinical trials in this specific population. An example of this includes the notion that future exercise-based interventions should focus on skeletal muscle mass and strength promoting protocols (e.g., resistance training) as opposed to selecting a protocol solely due to its ability to reduce adipose tissue. Future studies should also continue examining whether *APOE4* carriage affects the magnitude of exercise-induced neurocognitive benefits as well as the potential of sex-specific characteristics related to these adaptations.

## Data availability statement

The raw data supporting the conclusions of this article will be made available by the authors, without undue reservation.

## Ethics statement

The studies involving human participants were reviewed and approved by the Institutional Review Board for Human Subjects at the University of South Alabama (IRB#: 1760935-2). The patients/participants provided their written informed consent to participate in this study.

## Author contributions

JK and AN conceptualized the overarching research aims and developed the design of the methodology. MT and JK wrote the first draft of the manuscript. AB and NS collected and processed data under the supervision of AN. CF facilitated participant recruitment. BH provided the resources for the neurocognitive examinations and interpretations. JK, AN, BH, and CF acquired the financial support for the project leading to this publication. MT, AB, NS, CF, BH, AN, and JK reviewed and edited the manuscript. All authors read and the approved the final draft of the manuscript.
